# COVID-19 Outcomes and Genomic Characterization of SARS-CoV-2 Isolated From Veterans in New England States: Retrospective Analysis

**DOI:** 10.2196/31503

**Published:** 2021-12-17

**Authors:** Megan Lee, Ya Haddy Sallah, Mary Petrone, Matthew Ringer, Danielle Cosentino, Chantal B F Vogels, Joseph R Fauver, Tara D Alpert, Nathan D Grubaugh, Shaili Gupta

**Affiliations:** 1 Yale School of Medicine West Haven, CT United States; 2 Yale School of Public Health New Haven, CT United States; 3 VA Connecticut Healthcare System West Haven, CT United States

**Keywords:** infectious disease, COVID-19, epidemiology, veteran, outcome, sequencing, genetics, virus, United States, impact, testing, severity, mortality, cohort

## Abstract

**Background:**

Clinical and virologic characteristics of COVID-19 infections in veterans in New England have not been described. The average US veteran is a male older than the general US population. SARS-CoV-2 infection is known to cause poorer outcomes among men and older adults, making the veteran population an especially vulnerable group for COVID-19.

**Objective:**

This study aims to evaluate clinical and virologic factors impacting COVID-19 outcomes.

**Methods:**

This retrospective chart review included 476 veterans in six New England states with confirmed SARS-CoV-2 infection between April and September 2020. Whole genome sequencing was performed on SARS-CoV-2 RNA isolated from these veterans, and the correlation of genomic data to clinical outcomes was evaluated. Clinical and demographic variables were collected by manual chart review and were correlated to the end points of peak disease severity (based on oxygenation requirements), hospitalization, and mortality using multivariate regression analyses.

**Results:**

Of 476 veterans, 274 had complete and accessible charts. Of the 274 veterans, 92.7% (n=254) were men and 83.2% (n=228) were White, and the mean age was 63 years. In the multivariate regression, significant predictors of hospitalization (C statistic 0.75) were age (odds ratio [OR] 1.05, 95% CI 1.03-1.08) and non-White race (OR 2.39, 95% CI 1.13-5.01). Peak severity (C statistic 0.70) also varied by age (OR 1.07, 95% CI 1.03-1.11) and O2 requirement on admission (OR 45.7, 95% CI 18.79-111). Mortality (C statistic 0.87) was predicted by age (OR 1.06, 95% CI 1.01-1.11), dementia (OR 3.44, 95% CI 1.07-11.1), and O2 requirement on admission (OR 6.74, 95% CI 1.74-26.1). Most (291/299, 97.3%) of our samples were dominated by the spike protein D614G substitution and were from SARS-CoV-2 B.1 lineage or one of 37 different B.1 sublineages, with none representing more than 8.7% (26/299) of the cases.

**Conclusions:**

In a cohort of veterans from the six New England states with a mean age of 63 years and a high comorbidity burden, age was the largest predictor of hospitalization, peak disease severity, and mortality. Non-White veterans were more likely to be hospitalized, and patients who required oxygen on admission were more likely to have severe disease and higher rates of mortality. Multiple SARS-CoV-2 lineages were distributed in patients in New England early in the COVID-19 era, mostly related to viruses from New York State with D614G mutation.

## Introduction

### Background

Disease severity and outcomes of COVID-19 caused by SARS-CoV-2 vary among individuals who become infected, with several factors that have been suggested as predictors of mortality, including Charlson comorbidity index score, age, and BMI [1-6]. Other comorbidities such as cardiovascular disease, diabetes, and dementia are prevalent in patients who are hospitalized and can predict worse outcomes and complications following infection, but the current literature shows variable impacts of comorbidities in different populations [7-15].

Virologic characteristics have been suggested to impact the severity of disease, and concern has been raised about several variants being more transmissible or more lethal [16-18]. A dynamic nomenclature system (Pangolin) was developed to classify SARS-CoV-2 and identify lineages and mutations that could impact infectivity and virulence [19]. In the early era of COVID-19, there was significant concern about the D614G variant being more infectious, and SARS-CoV-2 with this mutation had been found to cause infections in New York State [17,20]. Evaluation of prevalence of this virus in the nearby states therefore became of substantial interest. Viral epidemiology and regional evaluation of viral variants, with their clinical correlations, are important to provide a full understanding of the disease.

### Study Rationale

Given the high variability and conflicting data in predicting who will have poor outcomes, assessment of specific populations is necessary to give providers the best clinical picture on their patients. The US veteran population is predominantly male (89%) with an average age of 58 years, compared to the general US population that has a median age of 38.5 years and a gender distribution of 98.2 males to every 100 females [21,22]. Both older age and male sex have been found to be independent predictors of greater disease severity and mortality in COVID-19 infections [8,13,23-26]. Evaluation of clinical outcomes among US veterans, a population predisposed to poorer outcomes because of these demographic variables, therefore, is important. Clinical outcomes of veterans with COVID-19 in New England and SARS-CoV-2 genomics have not been described. Outcome assessment based on unique demographic and clinical variables in people infected with novel viruses cannot be overemphasized. Given the high mutability of SARS-CoV-2, changing epidemiological trends over time, and known impact of virological factors on clinical outcomes, this study becomes even more important as it will provide an insight into this unique regional population with COVID-19 at a relevant time period in the evolution of the virus.

### Study Aims

We aimed to describe patient characteristics, comorbidities, and disease factors that impact patient outcomes and present data on the genomic composition of the SARS-CoV-2 infecting these patients.

## Methods

### Specific Objectives

This study aims to determine the clinical and virologic factors impacting outcomes in veterans with COVID-19.

### Study Design With Justification

We conducted a retrospective chart review to gather demographic and clinical variables as well as clinical outcomes. The Veterans Affairs (VA) health care system has electronic medical records that can be accessed to extract this information. We have conducted such chart reviews before to help inform management decisions based on predictors of outcomes [27].

### Study Setting

In 2020, the VA health care system in West Haven, Connecticut had been entrusted with testing for SARS-CoV-2 for all six VA health care centers in New England states (Connecticut, Massachusetts, Maine, New Hampshire, Rhode Island, Vermont). The virus, isolated from samples testing positive, was then sent for whole genome sequencing (WGS) under an agreement funded by the Centers for Disease Control and Prevention (CDC).

### Participants (Sample Size and Inclusion Criteria)

This study included all veterans who tested positive for COVID-19 from April 8, 2020, to September 16, 2020, at any of the six New England VA hospitals. Inclusion criteria included patients with accessible chart records and a diagnosis of COVID-19 based on one of three polymerase chain reaction–based tests: Xpert Xpress SARS-CoV-2 (Cepheid), Simplexa COVID-19 Direct Kit (DiaSorin), and Roche cobas 6800 system.

### Data Collection

We manually reviewed charts and recorded demographics (age, gender, race, BMI, long-term care [LTC] facility status, and state of residence when diagnosed with COVID-19). Comorbidities recorded were immunosuppression, dementia, diabetes mellitus, chronic kidney disease stage 3, chronic liver disease, coronary artery disease (CAD), heart failure, atrial fibrillation, chronic obstructive pulmonary disease, asthma, and active tobacco use. All data collection was retrospective after a diagnosis of COVID-19 had been confirmed. If chart review occurred while a veteran was hospitalized, the chart was again reviewed retrospectively after discharge from the hospital.

### Sample Collection and Handling

Handling of nasopharyngeal specimens or isolated virus was carried out by the VA Connecticut Healthcare System (VACHS) clinical laboratory as part of clinical care, following standardized Clinical Laboratory Improvement Amendments guidelines [28]. Our viral repository was populated by the positive test results of all New England veterans. The VACHS laboratory handled specimens, isolated the SARS-CoV-2 RNA, and shipped it for WGS to a non-VA laboratory. We obtained the details of the platform used to diagnose, the cycle threshold (Ct), and the date of test from the laboratory. Sequencing of viral genomes was conducted at the non-VA laboratory by our coauthors as follows.

### Genomic Sequencing and Phylogenetic Analysis

Whole virus genomes were sequenced (≥20x coverage depth across ≥70% of the genome) using the Illumina (n=238) and Nanopore (n=61) platforms. WGS was conducted on SARS-CoV-2 isolates with a Ct value <36 and provided near-complete or complete genome results where the Ct value was <30. Using BWA-MEM version 0.7.15 (GNU Project), we aligned reads to the Wuhan-Hu-1 reference genomes (GenBank MN908937.3). With iVar v1.2.1 (Andersen Lab), we trimmed sequencing adaptors and primer sequences, and called bases by simple majority (>50% frequency) at each site to generate consensus genomes. An ambiguous N was used when <10 reads were present at a site. We aligned consensus genomes with MAFFT (GNU Project) [29] and masked problematic sites [30]. We built a phylogenetic tree with IQTree (IQ-Tree) [31] using an HKY substitution model and 1000 bootstraps, visualized it with Python module baltic v0.1.5 (Python Software Foundation), and assigned lineages with Pangolin [19].

### Outcome Measures

Our categorical outcomes, also derived from manual chart review, were hospitalization status, mortality, and oxygen (O_2_) requirement within 24 hours of admission from manual chart review. We divided patients based on their peak disease severity (ordinal) into five categories depending on oxygenation requirements: (1) no O_2_ requirement, (2) 1 to 3 liters by nasal cannula (NC), (3) 4 to 6 liters by NC, (4) >6 liters O_2_ or noninvasive positive pressure ventilation, and (5) mechanical ventilation. Mortality was defined as death within 60 days from the date of diagnosis.

### Data Analysis

We used STATA v16 (StataCorp) for logistic regressions to predict our hospitalization and mortality, and ordinal logistic regression to predict peak disease severity. We first conducted a univariate analysis, then used significant variables from the univariate analysis (*P*<.05) to use in a multivariate model for each of our outcomes to assess the impact of several variables at once, which has been frequently used in COVID-19 literature [9,10,32-34]. Assumptions for logistic regressions (binary outcome, linearity, no outliers, and multicollinearity) were tested and met, with maximum variance inflation factors of 2. Genomic characteristics were reported descriptively.

### Ethical Considerations

The VACHS Institutional Review Board approved the creation and maintenance of a data repository of all veterans in New England diagnosed with COVID-19 and a viral repository of the SARS-CoV-2 RNA received from all six New England facilities. This study was conducted in accordance with the Declaration of Helsinki, keeping all private health information secure in approved secure folders behind a VA firewall. The RECORD (Reporting of Studies Conducted Using Observational Routinely-Collected Data) statement guidelines were used to maintain transparency in the reporting of this study [35].

## Results

### Participant Characteristics

Of 476 veterans in six New England states with confirmed SARS-CoV-2 during the study period, 274 had complete and accessible charts. Of 274 veterans, 93% (n=254) were men, 83% (n=228) were White, the mean age was 63 (SD 17.6) years, and over one-third resided were in LTC (n=92; Table 1). The most common comorbidities were CAD (n=74), diabetes (n=68), and tobacco use (n=62).

**Table 1:** Patient characteristics (n=274).

Demographics					Participants
Age (years), mean (SD)					62.9 (17.6)
**Gender, n (%)**					
	Male			254 (93)	
	Female			20 (7)	
**Race, n (%)**					
	White			228 (83)	
	Non-White			46 (17)	
BMI >30, n (%)					110 (40)
From LTC^a^, n (%)					92 (34)
**State, n (%)**					
	Connecticut			89 (32)	
	Massachusetts			150 (55)	
	Maine			4 (1)	
	New Hampshire			9 (3)	
	Rhode Island			20 (7)	
	Vermont			2 (1)	
**Comorbities, n (%)**					
	Immunosuppressed			10 (4)	
	Dementia			42 (15)	
	Diabetes			68 (25)	
	CKD^b^ 3			18 (7)	
	Chronic liver disease			32 (12)	
	**Chronic heart disease**			107 (39)	
		CAD^c^	74 (27)		
		Heart failure	29 (11)		
		Atrial fibrillation	40 (15)		
	**Chronic lung disease**			97 (35)	
		COPD^d^	44 (16)		
		Asthma	19 (7)		
		OSA^e^	55 (20)		
	Tobacco use			62 (23)	

^a^LTC: long-term care.

^b^CKD: chronic kidney disease.

^c^CAD: coronary artery disease.

^d^COPD: chronic obstructive pulmonary disease.

^e^OSA: obstructive sleep apnea.

### Rates and Predictors of Hospitalization, Peak Severity, and Mortality

**Figure 1:**
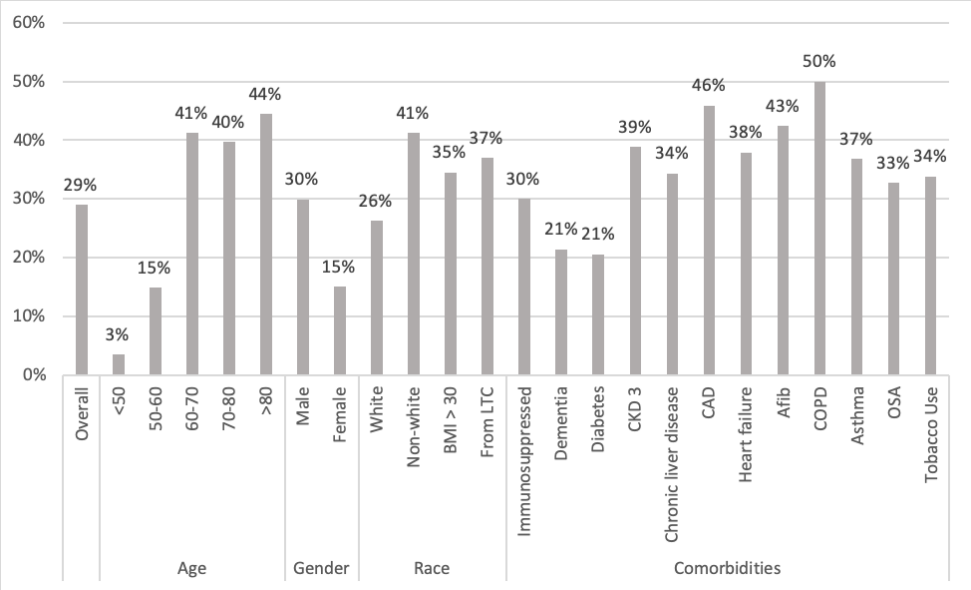
Percent of patients hospitalized based on patient demographics and comorbidities. CAD: coronary artery disease; CKD: chronic kidney disease; COPD: chronic obstructive pulmonary disease; LTC: long-term care; OSA: obstructive sleep apnea.

**Figure 2:**
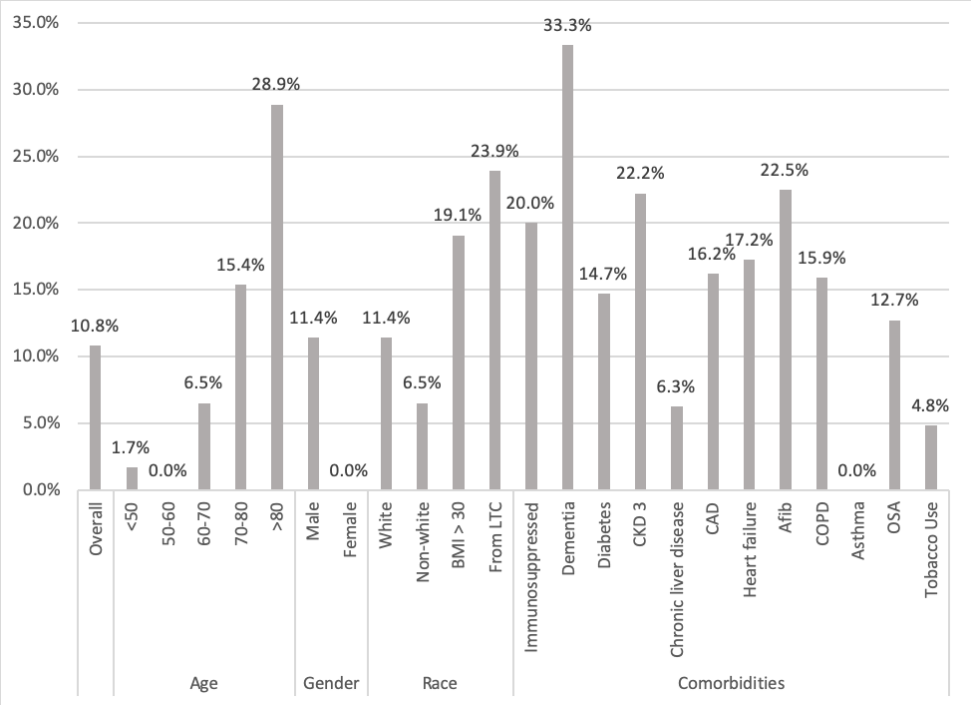
Percent of patients who died based on patient demographics and comorbidities. CAD: coronary artery disease; CKD: chronic kidney disease; COPD: chronic obstructive pulmonary disease; LTC: long-term care; OSA: obstructive sleep apnea.

Notably, 12% (n=33) of patients required O_2_ above their baseline home O_2_ requirement within 24 hours of admission, and 21% (n=58) of all patients required O_2_ support at some point during hospitalization. In terms of peak severity, 79% (n=216) required only room air, 11% (n=30) required 1 to 3 liters O_2_, 4.0% (n=11) required 4 to 6 liters O_2_, 3.6% (n=10) required >6 liters O_2_ or noninvasive positive-pressure ventilation, and 2.2% (n=6) were intubated. The hospitalization rate was 29% (n=79; Figure 1), and the overall mortality rate was 11% (n=30; Figure 2).

Univariate regression analysis results are reported in Table 2. In the multivariate regression (Table 2), significant predictors of hospitalization (C statistic 0.75) were age (odds ratio [OR] 1.05, 95% CI 1.03-1.08) and non-White race (OR 2.39, 95% CI 1.13-5.01; Table 2). Peak severity (C statistic 0.70) also varied by age (OR 1.07, 95% CI 1.03-1.11) and O_2_ requirement on admission (OR 45.7, 95% CI 18.79-111). Mortality (C statistic 0.87) was predicted by age (OR 1.06, 95% CI 1.01-1.11), dementia (OR 3.44, 95% CI 1.07-11.1), and O_2_ requirement on admission (OR 6.74, 95% CI 1.74-26.1). In other words, for every year increase in age, the odds of hospitalization increased by 5%, peak severity increased by 7%, and mortality increased by 6%.

**Table 2:** Univariate and multivariate regression analysis of factors that predict hospitalization, peak severity, and death.

		Hospitalization		Peak severity		Death	
		Unadjusted OR^a^ (95% CI)	*P* value	Unadjusted OR (95% CI)	*P* value	Unadjusted OR (95% CI)	*P* value
**Univariate regression**							
	Age	1.06 (1.03-1.08)	*<.001^b^*	1.06 (1.04-1.09)	*<.001*	1.1 (1.06-1.14)	*<.001*
	Female gender	0.41 (0.12-1.45)	.17	0.42 (0.95-1.9)	.26	N/A^c^	N/A
	Non-White race	1.97 (1.02-3.8)	*.04*	1.3 (0.6-2.6)	.55	0.54 (0.16-1.9)	.33
	From long-term facility	1.78 (1.04-3.07)	*.04*	2.8 (1.5-5.1)	*.001*	7.9 (3.2-19.2)	*<.001*
	BMI <30	1.52 (0.9-2.7)	.14	1.1 (0.6-2)	.77	5.8 (2.1-15.9)	*.001*
	Dementia	0.63 (0.29-1.4)	.25	1.6 (0.7-3.3)	.26	7.2 (3.2-16.6)	*<.001*
	COPD^d^	3.04 (1.6-5.9)	*.001*	1.5 (0.74-3.2)	.25	1.8 (0.7-4.5)	.22
	Heart failure	1.6 (0.7-3.5)	.25	1.8 (0.8-4.0)	.18	1.9 (0.67-5.5)	.23
	CAD^e^	2.93 (1.66-5.2)	*<.001*	2.1 (1.2-3.9)	*.02*	2.1 (0.9-4.6)	.07
	Atrial fibrillation	2.05 (1.03-4.1)	*.04*	3 (1.4-5.9)	*.002*	3.1 (1.3-7.4)	*.01*
	Hospitalization	N/A	N/A	N/A	N/A	2.58 (1.18-5.65)	*.02*
	O_2_ on admission	N/A	N/A	46.2 (19.9-107.3)	*<.001*	4.34 (1.77-10.6)	*.001*
**Multivariate regression**							
	Age	1.05 (1.03-1.08)	*<.001*	1.07 (1.03-1.11)	*<.001*	1.06 (1.01-1.11)	*.02*
	Non-White race	2.39 (1.13-5.01)	*.02*	N/A	N/A	N/A	N/A
	From long-term facility	0.70 (0.36-1.38)	.31	1.18 (0.55-2.55)	.67	2.0 (0.58-6.88)	.27
	BMI <30	N/A	N/A	N/A	N/A	2.95 (0.81-10.75)	.10
	Dementia	N/A	N/A	N/A	N/A	3.44 (1.07-11.1)	*.04*
	COPD	1.73 (0.84-3.35)	.14	N/A	N/A	N/A	N/A
	CAD	1.44 (0.75-2.81)	.27	0.71 (0.32-1.55)	.39	0.57 (0.209-1.57)	.28
	Atrial fibrillation	0.984 (0.45-2.16)	.97	1.11 (0.46-2.64)	.82	1.25 (0.42-3.69)	.69
	Hospitalization	N/A	N/A	N/A	N/A	1.36 (0.40-4.65)	.63
	O_2_ on admission	N/A	N/A	45.7 (18.79-111)	*<.001*	6.74 (1.74-26.1)	*.006*

^a^OR: odds ratio.

^b^Italics indicate that *P*<.05 (exact values reported).

^c^N/A: not applicable.

^d^COPD: chronic obstructive pulmonary disease.

^e^CAD: coronary artery disease.

### Genomic Characteristics

**Figure 3:**
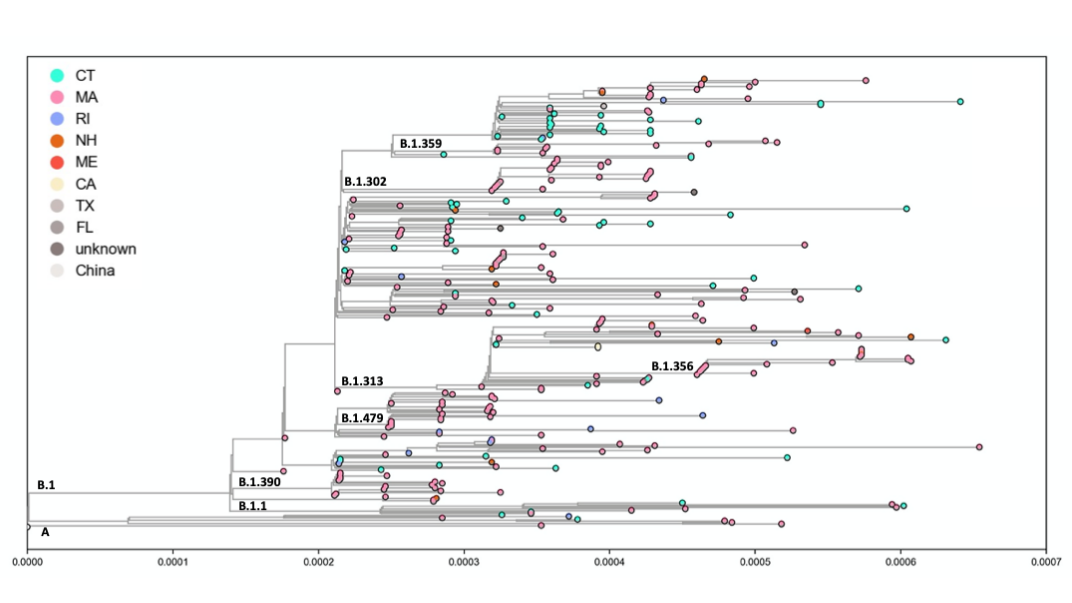
Maximum likelihood tree of genomes.

For the genomic characteristics, among the 476 patients, 299 patients’ genomes had adequate coverage for analysis. We found the majority of our specimens (154/299, 51.5%) were from SARS-CoV-2 lineage B.1 or a sublineage of B.1 (eg, B.1.302, B.1.303, B.1.356; 137/299, 46%), all of which are defined by D614G substitution (Figure 3, Multimedia Appendix 1). Only 2.4% (7/299) were from the lineage A that lack the D614G mutation. There were 41 different SARS-CoV-2 lineages detected in our cohort, and we did not have the power to test for clinical correlates. Our sequencing data does inform us that the outcomes presented in this VA cohort are dominated by the impacts of the B lineage D614G mutation.

## Discussion

### Principal Findings

Our study found that in a cohort of veterans with an average age of 63 years and a high comorbidity burden, age was significantly associated with risk of hospitalization, peak disease severity, and mortality. O_2_ requirement upon admission correlated with peak disease severity and mortality, while dementia was an additional factor associated with higher mortality. The CDC provides a list of chronic medical conditions (May 2021) that predispose individuals to severe illness from SARS-CoV-2 infection [6]. Based on this list, >75% of US adults fall under a high-risk category [36], therefore making it important to have select populations evaluated for uniquely applicable risk factors. Veterans are a unique cohort because of advanced age on average [22] and more comorbidities. Understanding clinical factors that impact outcomes in veterans will help health care providers risk-stratify patients with similar demographic profiles, and future research should explore the impact of new treatments and vaccination on outcomes. The predominance of B lineage D614G in our study specimens provided valuable insight into the pace of epidemiological trend and the evolution of the virus early in the COVID-19 era through the New England region.

### Comparison With Prior Studies

Many COVID-19 studies have found age to be a predictor of worse outcomes [3,37-40]. In our study, age was a significant predictor for all of the studied outcomes and was a confounder for other variables. Accordingly, LTC status predicted all three of our outcomes on univariate analysis but not on multivariate analyses, possibly because LTC units tend to have older residents. Earlier in the COVID-19 pandemic, residents of nursing homes had higher rates of infection and severe illness and mortality [41]. Our study shows disease outcomes were not impacted by their residence status after adjusting for age.

Concurrent work from our group suggests that O_2_ requirement within 24 hours of admission predicts poor outcomes in veterans, which has helped inform the triage guidelines at our health care system. This is an important finding because other ways of determining oxygenation status can frequently change and thus become difficult for clinicians to use in practice [22].

Our study supports data from previous reports that non-White patients in the United States are at increased risk of hospitalization but have similar peak severity and mortality outcomes [42,43]. Many studies have shown that minorities often have delays in seeking care, causing higher risk of hospitalization when they do seek care [44,45]. This may explain the outcomes in our study. It is critical to continue ongoing efforts to combat medical inequities and target prevention efforts and education to communities and racial groups most affected by COVID-19.

After adjusting for age and other comorbidities, we found that patients with dementia had a higher risk of death. This is similar to other studies on patients with COVID-19 and dementia [37,46,47]. This may be explained by a host of biological factors but also may be a result of the inability to self-report symptoms. This finding emphasizes the importance of extra care and monitoring required when approaching a patient with dementia.

### Limitations

Limitations of this study include the smaller sample size. Furthermore, our study is specific to veterans, which is a largely male and older cohort, and results may not therefore be generalizable. The time period of this study was prior to established medical therapies for COVID-19, and our reported outcomes are likely worse than expected today. Strengths of our study include its comprehensive scope, wide geographic range, manual chart review process allowing for the capturing of all comorbidities and oxygenation parameters that may not be available otherwise in a database, and multivariate analysis of many potential risk factors.

### Conclusion

Our study found that in an older cohort of veterans from the six New England states with a high comorbidity burden, age was the single strongest predictor of hospitalization, peak severity, and mortality. Non-White veterans were more likely to be hospitalized, and patients who required oxygen on admission were more likely to have severe disease and higher rates of mortality. Furthermore, patients with dementia were more likely to die. Multiple genomic variants of SARS-CoV-2 were distributed in patients in New England early in the COVID-19 era, mostly from a B.1 sublineage with the spike D614G mutation.

## Appendix

Multimedia Appendix 1 Lineages of genomes.

## Abbreviations

CAD: coronary artery disease
CDC: Centers for Disease Control and Prevention
Ct: cycle threshold
LTC: long-term care
NC: nasal cannula
OR: odds ratio
REPORT: Reporting of Studies Conducted Using Observational Routinely-Collected Data
VA: Veterans Affairs
VACHS: VA Connecticut Healthcare System
WGS: whole genome sequencing
