# Supplementary material for: COVID-19 Outcomes and Genomic Characterization of SARS-CoV-2 Isolated From Veterans in New England States: Retrospective Analysis
Source: JMIRx Med. 2021 Dec 17;2(4):e31503. doi: 10.2196/31503 (PMC8722526; doi:10.2196/31503)
Supplement: Multimedia Appendix 1 [file xmed_v2i4e31503_app1.docx]

Multimedia Appendix 1. Lineages of genomes

| Lineage | Count | Percent |
| --- | --- | --- |
| A.1 | 3 | 1.0% |
| A.2 | 2 | 0.7% |
| A.3 | 2 | 0.7% |
| B | 1 | 0.3% |
| B.1 | 154 | 51.5% |
| B.1.1.10 | 1 | 0.3% |
| B.1.1.113 | 1 | 0.3% |
| B.1.1.128 | 1 | 0.3% |
| B.1.1.163 | 1 | 0.3% |
| B.1.1.192 | 1 | 0.3% |
| B.1.1.225 | 1 | 0.3% |
| B.1.1.307 | 1 | 0.3% |
| B.1.104 | 4 | 1.3% |
| B.1.108 | 1 | 0.3% |
| B.1.110.3 | 1 | 0.3% |
| B.1.2 | 3 | 1.0% |
| B.1.243 | 1 | 0.3% |
| B.1.268 | 1 | 0.3% |
| B.1.280 | 2 | 0.7% |
| B.1.302 | 26 | 8.7% |
| B.1.313 | 19 | 6.4% |
| B.1.319 | 1 | 0.3% |
| B.1.320 | 1 | 0.3% |
| B.1.331 | 1 | 0.3% |
| B.1.356 | 18 | 6.0% |
| B.1.359 | 8 | 2.7% |
| B.1.36 | 1 | 0.3% |
| B.1.369 | 3 | 1.0% |
| B.1.382 | 1 | 0.3% |
| B.1.385 | 1 | 0.3% |
| B.1.390 | 16 | 5.4% |
| B.1.403 | 1 | 0.3% |
| B.1.413 | 1 | 0.3% |
| B.1.448 | 2 | 0.7% |
| B.1.448 | 1 | 0.3% |
| B.1.479 | 10 | 3.3% |
| B.1.509 | 2 | 0.7% |
| B.1.517 | 1 | 0.3% |
| B.1.521 | 1 | 0.3% |
| B.35 | 1 | 0.3% |
| N.1 | 1 | 0.3% |
